# A Comparison Between AMS 700 and Coloplast Titan: A Systematic Literature Review

**DOI:** 10.7759/cureus.11350

**Published:** 2020-11-05

**Authors:** Elias Atri, Vivian Wong, Noel C Barengo, Alan M Nieder, Alan S Polackwich

**Affiliations:** 1 Urology, Florida International University Herbert Wertheim College of Medicine, Miami, USA; 2 Translational Medicine, Florida International University Herbert Wertheim College of Medicine, Miami, USA; 3 Urology, Mount Sinai Medical Center, Miami, USA

**Keywords:** penile implants, erectile dysfunction, penile prosthesis

## Abstract

There are only two three-piece inflatable penile prostheses (IPP) available to patients in the American market: the AMS (American Medical Systems) 700^TM^ series (Boston Scientific, Massachusetts) and the Coloplast Titan® series (Coloplast, Minnesota), and data comparing the two are scant. The aim of our study was to summarize the current scientific evidence comparing the two.

A systematic literature review was conducted on PubMed. A 10-year filter was placed to include only studies published after Coloplast launched the Titan Touch® release pump. Eligibility criteria included articles discussing specifically the AMS 700^TM^ and Coloplast Titan® models. Further searches for studies on patient/partner satisfaction were conducted. Abstracts were reviewed to include studies focusing specifically on the models we are studying and studies on patient satisfaction using the Erectile Dysfunction Inventory of Treatment Satisfaction (EDITS) questionnaire.

The Coloplast device demonstrated slightly greater resistance to the stimulated forces of penetration and gravity. Coloplast implants coated with vancomycin/gentamicin had the highest infection rate followed by the AMS penile prosthesis and the rifampin/gentamicin coating had the lowest infection rate. Prosthesis durability and survival were similar between both brands. Overall satisfaction was high but comparisons are inconsistent.

The literature is inconclusive about which device is superior. We suggest randomized, multicenter, prospective studies to help further elucidate the highlights of each product.

## Introduction and background

Erectile dysfunction (ED) is a recurrent or consistent inability to acquire or sustain an erection of sufficient rigidity and duration for sexual intercourse [1]. ED has been estimated to affect approximately 5%-20% of men. Differences in the definitions of ED, as well as methodological differences, may explain the variety in reported prevalence rates [2]. The Multinational Men’s Attitudes to Life Events and Sexuality study identified the overall prevalence of ED to be 16% and found the prevalence to be 20% in the United States. The prevalence also increased with age and other comorbid medical conditions such as cardiovascular disease, hypertension, dyslipidemia, and depression. Of the men in the study, 58% had actively sought medical attention for ED and only 16% were currently being treated with oral phosphodiesterase-5 inhibitor (PDE-5) therapy [3].

As per evidence-based medicine, treatment for ED involves identifying the underlying etiology (including drugs such as antidepressants and antihypertensive medications), identifying and treating cardiovascular risk factors, and then initiating medical therapy with the first-line agents, PDE-5 inhibitors. If PDE-5 inhibitors are contraindicated or ineffective, vacuum devices, penile self-injectable drugs, and intraurethral alprostadil are second-line therapy [1]. The surgical implantation of a penile prosthesis is indicated for men who cannot use or have not responded to first- and second-line therapies and inflatable penile prostheses (IPP) account for the majority of these implants [4]. However, the American Urological Association's clinical guidelines now suggest that with proper counseling and shared decision-making, men can choose what their initial therapy will be.

Currently, the only two three-piece IPPs manufactured in the United States are the AMS 700^TM^ series prostheses (Boston Scientific, Massachusetts) and the Coloplast Titan® prostheses (Coloplast, Minnesota) [5]. There are three variations in the AMS 700^TM^ line: the AMS 700^TM^ LGX, the AMS 700^TM^ CX, and the AMS 700^TM^ CXR. Both the AMS 700^TM^ series and the Coloplast Titan® are three-component prostheses that consist of two cylinders implanted into the corpora cavernosa, a pump placed in the scrotum, and a reservoir filled with saline placed in the space of Retzius or the high submuscular space. The AMS devices are available with an outer layer of Inhibizone^TM^ made of rifampin and minocycline to prevent infection. These devices also have the Momentary Squeeze Pump^TM^, which allows users to press the button once and then squeeze the cylinders (instead of having to do so simultaneously), making deflation easier, and it also has a lockout valve that prevents auto-inflation. Coloplast has a hydrophilic coating that can absorb the antibiotics into which it is immersed and a Titan® Touch release pump that requires only a compression of the release pump to allow for complete cylinder deflation [6].

Given the difference in device design, we aimed to review the current scientific literature comparing these two IPPs, which includes a biomechanical comparison, infection rates, the use of IPPs in Peyronie’s disease (PD), and patient and partner satisfaction.

## Review

Protocol

This is a systematic review of the literature concerning the comparison of the Coloplast Titan® and the AMS 700^TM^, as well as patient and partner satisfaction with each IPP. The methods and reporting follow the Preferred Reporting Items for Systematic Reviews and Meta-Analyses (PRISMA) statement [7].

Eligibility Criteria

The inclusion criterion for the first search was articles published in the last 10 years. This 10-year filter was placed to include only studies published after Coloplast launched the Titan® Touch release pump for easier deflation in 2008 [8]. Additional eligibility criteria included articles discussing specifically the AMS 700^TM^ and Coloplast Titan® models. For the second and third searches, studies of IPP use for ED were eligible and those studying IPPs implanted for PD were excluded. Additionally, abstracts were reviewed to include studies focusing specifically on the models we are studying and studies on patient satisfaction using the Erectile Dysfunction Inventory of Treatment Satisfaction (EDITS) questionnaire.

Information Sources

**Figure 1:**
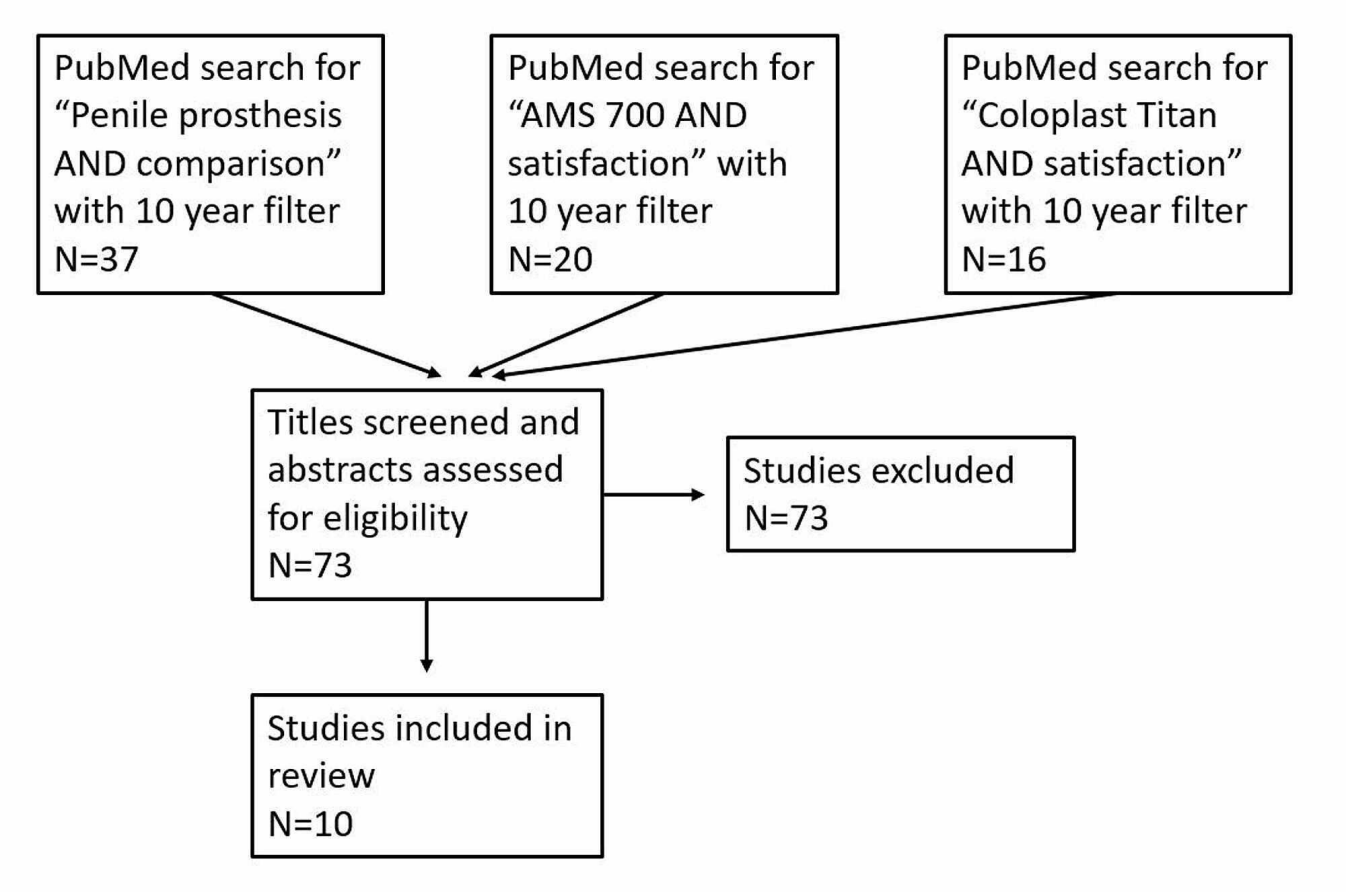
PRISMA diagram PRISMA: Preferred Reporting Items for Systematic Reviews and Meta-Analyses

Table 1 presents the keywords used and the results of the literature search. PubMed was searched using the term “penile prosthesis AND comparison.” This yielded 37 articles. After reviewing abstracts for inclusion criteria, six articles were included. The search term “Coloplast Titan® AND satisfaction” yielded 16 articles, out of which two were included. A similar search using “AMS 700^TM^ AND satisfaction” yielded 20 articles, out of which two were included. Figure 1 presents the PRISMA diagram for our review. Table 2 presents a summary of the study designs and characteristics.

**Table 1:** Keywords and results of the literature search

Search Term	Number of Articles	Number Included
Penile prosthesis AND comparison	37	6 (Otero, Al Ansari, Morgado, Chung, Wallen, Dhabuwala)
Coloplast Titan AND satisfaction	16	2 (Lindeborg, Garrido)
AMS 700 AND satisfaction	20	2 (Negro, Vitarelli)

**Table 2:** Summary of study designs and characteristics

Author	Study Setting	Study Design	Sample Size	Specific IPP Studied
Al Ansari et al. [9]	Qatar, 2013	Cross-sectional study	100	Coloplast Titan and AMS 700
Wallen et al. [10]	Florida, 2018	Biomechanical evaluation	-	Coloplast Titan and AMS 700
Dhabuwala et al. [11]	Michigan, 2011	Review	339	Coloplast Titan and AMS 700
Chung et al. [12]	Canada, 2013	Review with cross-sectional survey	138	Coloplast Titan and AMS 700
Lindeborg et al. [13]	Denmark, 2014	Prospective cohort study with survey	26	Coloplast Titan
Garrido et al. [14]	Spain, 2015	Review with cross-sectional survey	100	Coloplast Titan
Negro et al. [15]	Italy, 2016	Prospective cohort study	36	AMS 700
Vitarelli et al. [16]	Italy, 2013	Review with cross-sectional survey	80	AMS 700
Otero et al. [17]	Spain, 2017	Retrospective non-randomized intervention study	248	Coloplast Titan and AMS 700
Morgado et al. [18]	Portugal, 2018	Cross-sectional survey	55	Coloplast Titan and AMS 700

Study Selection

Results from the search were saved in a Microsoft Excel (Microsoft Corporation, Redmond, WA), spreadsheet, and duplicates were removed. Two reviewers (EA and VW) independently reviewed titles and abstracts. Disagreements were solved by consensus between them.

Data Collection and Synthesis of Results

Data were collected in an Excel sheet and information included study setting, study design, sample size, and specific IPP studied. The key results from selected studies were summarized in text.

Ethical Statement

This is a systematic review and meta‐analysis of published and open information. No human subjects were involved in this project. This study was therefore classified as non-human subject research and no IRB approval was necessary.

Literature review

Comparison of Biomechanics

A study by Al Ansari et al. aimed to look at the effects of axial rigidity on patient satisfaction rates in different types of penile prosthetics. One-hundred patients were surveyed for satisfaction after penile prosthesis implant surgery and the digital inflection rigometer (DIR) was used to assess axial rigidity. Partners of patients were also surveyed for satisfaction after implantation. The study assessed various types of IPPs, including Coloplast Titan® and AMS 700^TM^ CX. Specifically, more patients received AMS 700^TM^ CX (N=42) versus Coloplast Titan® (N=13) and Coloplast Titan OTR® (N=15). Mean DIR for the AMS 700^TM^ CX was 985 and for the Coloplast Titan® and Coloplast Titan OTR®, it was 1068 and 953, respectively. The dissatisfaction rate of AMS 700^TM^ CX was lower than both Titan® with a percentage of 2.4% versus 7.7% and 6.6%. Therefore, even with the slightly increased axial rigidity in Titan®, 700^TM^ CX showed a lower dissatisfaction rate [9].

Wallen et al. looked at the biomechanical properties of three different penile prosthetics, AMS CX and AMS LGX, which are both part of the AMS 700^TM^ line, and Coloplast Titan®. They specifically studied the axial load, kink formation, horizontal stiffness, and resistance to three-point flexure testing on human cadavers. Coloplast Titan® had slightly significantly increased rigidity in horizontal load testing, and increased rigidity in the longest phallus and the phallus with mild PD, as compared to both the AMS CX and AMS LGX. However, in the shortest phallus, the AMS CX had better rigidity [10].

Infection Rates

Dhabuwala et al. studied the infection rates between different types of penile implants and different antibiotic preparations of the devices. Coloplast Titan® penile implants coated with vancomycin/gentamicin, Coloplast Titan® penile implants coated with rifampin/gentamicin, and Inhibizone^TM^-impregnated AMS penile implants were all compared. The implants coated with vancomycin/gentamicin had an infection rate of 4.4%, the Inhibizone^TM^-impregnated AMS penile implants had an infection rate of 1.3%, and none of the implants coated with rifampin/gentamicin developed an infection. The study did not make any claims about the superiority of certain types of penile implants over others but did suggest that all Coloplast Titan® penile implants be prepared with rifampin/gentamicin [11].

Comparison of IPP Use in the Treatment of PD

Simultaneous manual penile remodeling and IPP implantation allow for a single procedure that corrects penile curvature and ED. Chung et al. evaluated clinical outcomes and patient satisfaction in AMS 700^TM^ and Coloplast Titan® use in patients with PD and ED. A clinical database review and a prospective telephone survey were used in a single-center retrospective review. No statistically significant difference in device survival was found. Both devices also provided similar penile strengthening without the need for revision surgery [12].

Patient and Partner Satisfaction

Lindeborg et al. evaluated patient satisfaction in 26 patients who underwent Titan® implantation through an EDITS questionnaire. Eighty-five percent reported being satisfied, 92% would recommend an implant to someone with a similar medical condition, and 72% believed their partner was satisfied with the implant [13]. Garrido et al. conducted a retrospective review of Titan® prosthesis performance and patient and partner satisfaction using a modified EDITS questionnaire. Overall satisfaction was 90% and 84% amongst patient and partner, respectively, in modified EDITS [14].

Negro et al. directly studied satisfaction after the implantation of the AMS 700^TM^ LGX. They found the International Index of Erectile Function (IIEF) scores at six and 12 months were significant for the desired domain (P = 0.0001) and for overall satisfaction (P = 0.002); however, mean EDITS scores at six and 12 months were not significantly improved. The greatest improvement in satisfaction was noted after one year post-surgery, which the authors suggested may be due to both the learning curve as well as the behavioral and psychosexual adjustment to the IPP [15].

Vitarelli et al. also studied long-term satisfaction with the implantation of AMS 700^TM^ CX/CXR as measured by IIEF and the EDITS questionnaire. They found high levels of satisfaction, with 87.7% of patients reporting satisfaction with implantation with 77.6% prosthesis survival rate at 10 years post-surgery [16].

Otero et al. compared patient and partner satisfaction between the two models. An 11-question, validated, but modified, questionnaire was used to evaluate patient satisfaction and a non-validated two-item questionnaire was given to the partner in a retrospective, multicenter, non-randomized study. Two-hundred forty-eight patients participated and 207 couples completed the questionnaire. Out of the 248 patients, 194 received the 700^TM^ CX and 54 received the Titan®. Overall satisfaction was very high for both prostheses, and both showed reliability for sexual intercourse. Significant differences, however, were found in three questions. More patients were satisfied with the 700^TM^ CX (P=0.0001). Only 4% with the 700^TM^ CX were dissatisfied with the deflation compared to 24% with the Titan® (P=0.0031). No patient with the Titan® took longer than six months to optimal management, defined as the use of the device for sexual intercourse with no help or with the help of his partner [17].

Morgado also compared Coloplast Titan® with AMS 700^TM^ CX. They assessed 55 patients who received either one of the prostheses using the EDITS questionnaire. The study found that there was no difference in satisfaction between patients with Titan® and patients with AMS 700^TM^ CX [18].

Discussion

Summary of Evidence

Biomechanical studies indicate slightly increased axial rigidity in the Titan® model, although this may not be clinically significant. Otherwise, kink formation, horizontal stiffness, and resistance to three-point flexure were comparable. Infection rates were slightly higher in implants coated with vancomycin/gentamicin at 4.4%, compared to Inhibizone^TM^-impregnated AMS penile implants (1.3%), and rifampin/gentamicin preparations (0%). In PD, sources indicate that both implants offer good outcomes with no difference in device outcomes. Overall satisfaction with IPPs is high but differences between the two devices are inconsistent. Lindeborg et al., 2013, report an 85% satisfaction rate with the Titan® implant and a 92% recommendation rate. Otero et al., 2017, however, found more patients satisfied with the 700^TM^ CX. The results are summarized in Table 3.

**Table 3:** Summary of results

	Author	Results
Comparison of biomechanics	Al Ansari et al. [9]	Slightly increased axial rigidity in Titan, but 700 CX showed a lower dissatisfaction rate.
	Wallen et al. [10]	Coloplast Titan had increased rigidity in horizontal load testing and in the longest phallus and the phallus with mild PD. In the shortest phallus, the AMS CX had better rigidity.
Infection rates	Dhabuwala et al. [11]	No claims about the superiority of certain types of penile implants over others but did suggest that all Coloplast Titan penile implants be prepared with vancomycin/gentamicin.
Comparison of IPP use in the treatment of PD	Chung et al. [12]	No statistically significant difference in device survival was found. Both devices also provided similar penile strengthening without the need for revision surgery.
	Patient and partner satisfaction	Lindeborg et al. [13]	85% reported being satisfied with Coloplast Titan, 92% would recommend an implant to someone with a similar medical condition, and 72% believed their partner was satisfied with the implant
Negro et al. [15]		The greatest improvement in satisfaction was noted after 1-year post-AMS 700 LGX implantation.
Vitarelli et al. [16]		87.7% of patients reported satisfaction with AMS 700 implantation with 77.6% prosthesis survival rate at 10 years post-surgery.
Otero et al. [17]		More patients were satisfied with the 700CX. Only 4% with the 700 CX were dissatisfied with the deflation compared to 24% with the Titan. No patient with the Titan took longer than 6 months to optimal management.
	Morgado et al. [18]	No difference in satisfaction between patients with Titan and patients with AMS 700 CX.

Limitations

One limitation of our project is the nature of this study. Since this is a literature review, we must rely on information from other studies where we are unable to account for bias. Additionally, some studies had a low number of patients, different patient cohorts, different surgical methods, and were predominantly retrospective. Furthermore, the selection of these studies for review is potentially biased and limited since we only looked at studies published in English and available through PubMed published within the last 10 years. Furthermore, from our search, we found no international evidence for the superiority of each of these IPPs.

Additionally, Kramer et al., 2010, compared preoperative expectations to postoperative satisfaction in 21 patients that underwent IPP surgery. Lower preoperative expectations correlated almost linearly to higher postoperative satisfaction scores (R=-0.489; P=0.0245). This indicates that realistic expectations lead to higher postoperative satisfaction [19]. Preoperative expectations may play an important role in postoperative satisfaction, as Kramer et al. suggest. Since the comparability of the preoperative expectations for these studies is unknown, this could limit our conclusions drawn from the studies. Additionally, the etiology of ED was unknown in the satisfaction studies, and this could play an important factor in the satisfaction with IPPs.

Importance and Relevance

The importance of our study is that, to our knowledge, this is the first literature review that synthesizes key evidence comparing AMS 700^TM^ and Coloplast Titan®. With our study, we look at various components of these two brands of IPPs in the treatment of ED and access the quality, satisfaction, and outcomes.

## Conclusions

Inflatable penile prostheses have been used successfully for ED not responsive to less invasive therapy. Both the AMS 700^TM^ series and the Coloplast Titan® have three components, infection prevention mechanisms, and valves that make deflation easier for the user as well as prevent auto-inflation. We found little substantial difference between the two types of IPPs, with studies showing inconsistent minor superiority of one over the other. We recommend surgeons use their own clinical judgment and preference when choosing the right IPPs to use. Preoperative expectations may play an important role and further research controlling for this variable is necessary. Lastly, prospective randomized multicenter trials may ultimately determine the ideal prostheses for specific patients.
